# Recent Advances on Pt-Free Electro-Catalysts for Dye-Sensitized Solar Cells

**DOI:** 10.3390/molecules26175186

**Published:** 2021-08-26

**Authors:** Yi-June Huang, Prasanta Kumar Sahoo, Dung-Sheng Tsai, Chuan-Pei Lee

**Affiliations:** 1 Department of Chemical Engineering, Stanford University, Stanford, CA 94305, USA; 2 Department of Mechanical Engineering, Siksha ‘O’ Anusandhan, Deemed to Be University, Bhubaneswar 751030, India; prasantakumarsahoo@soa.ac.in; 3 Department of Electronic Engineering, Chung Yuan Christian University, Taoyuan City 32023, Taiwan; 4 Department of Applied Physics and Chemistry, University of Taipei, Taipei 10048, Taiwan

**Keywords:** carbon, conductive polymers, counter electrode, dye-sensitized solar cells, hybrids compound, metal compound, Pt-free electro-catalyst

## Abstract

Since Prof. Grätzel and co-workers achieved breakthrough progress on dye-sensitized solar cells (DSSCs) in 1991, DSSCs have been extensively investigated and wildly developed as a potential renewable power source in the last two decades due to their low cost, low energy-intensive processing, and high roll-to-roll compatibility. During this period, the highest efficiency recorded for DSSC under ideal solar light (AM 1.5G, 100 mW cm^−2^) has increased from ~7% to ~14.3%. For the practical use of solar cells, the performance of photovoltaic devices in several conditions with weak light irradiation (e.g., indoor) or various light incident angles are also an important item. Accordingly, DSSCs exhibit high competitiveness in solar cell markets because their performances are less affected by the light intensity and are less sensitive to the light incident angle. However, the most used catalyst in the counter electrode (CE) of a typical DSSC is platinum (Pt), which is an expensive noble metal and is rare on earth. To further reduce the cost of the fabrication of DSSCs on the industrial scale, it is better to develop Pt-free electro-catalysts for the CEs of DSSCs, such as transition metallic compounds, conducting polymers, carbonaceous materials, and their composites. In this article, we will provide a short review on the Pt-free electro-catalyst CEs of DSSCs with superior cell compared to Pt CEs; additionally, those selected reports were published within the past 5 years.

## 1. Introduction

Our civilization was born from fire and electricity energy. Everything in human life relies on fire and electricity energy for development. The energy requirements sharply increase with the growth of industry and population day by day [1,2,3,4]. At the same time, air pollution and greenhouse gas emissions are dramatically raising too. The development of a sustainable energy resource is an important and urgent challenge for people. Nowadays, solar cells are considered as one of the most promising energy suppliers. This idea is based on solar (sun) radiation of about 120,000 terawatts on Earth’s surface, which is approximately thousands of times of current energy consumption for a year [3,4]. Solar cells can directly converse photons (solar radiation) to electrons (electricity). In this regard, solar cells are important for the development of sustainable energy resources in our civilization.

**Figure 1:**
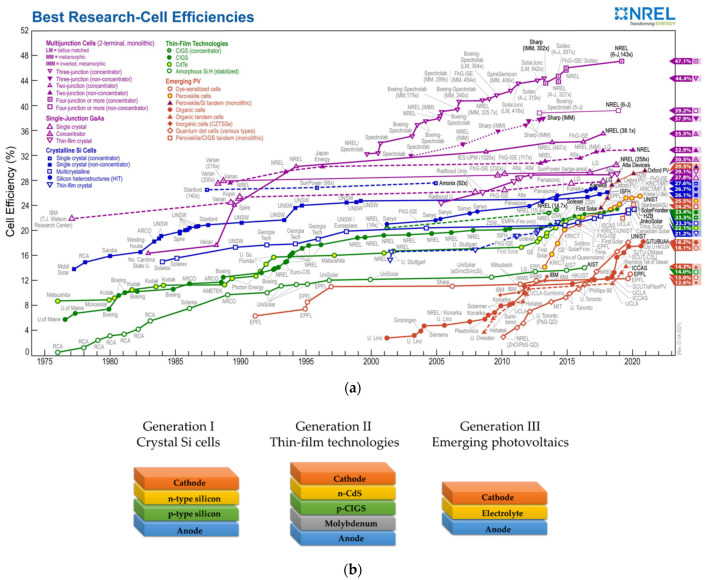
(**a**) A chart of the highest confirmed conversion efficiencies for research cells for a range of photovoltaic technologies plotted from 1976 to the present. (from the national laboratory, NERL, of the U.S. Department of Energy, https://www.nrel.gov/pv/cell-efficiency.html, accessed on 22 March 2021) (**b**) The illustration of three generations of solar cells. [10].

Solar cells have been classified into three generations with various performances and characters, as shown in Figure 1. The first generation is crystal Si cells and includes concentrator single crystal, non-concentrator single crystal, multi-crystal, silicon heterostructure, and thin-film crystal. The second generation is thin-film technologies and contains CdTe, GaAs, CdS, CZTS (CuZnTiSe), CIGS (CuInGaSe), and amorphous Si. Finally, the third generation is emerging photovoltaics and includes dye-sensitized solar cells (DSSCs), perovskite cells, perovskite/Si cells, organic cells, and CZTSSe (inorganic) cells [4,5,6,7,8]. Among them, the DSSCs fascinate scientists because they offer easy fabrication, economic products, are environmentally friendly, and provide outstared performance under ambient light [7,9,10,11].

**Figure 2:**
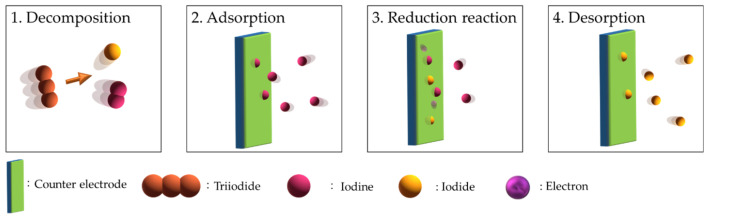
The schematic diagram of the electrochemical regeneration reactions of I_3_^−^ ions at a counter electrode.

A typical DSSC consists of a photoanodic electrode (anode), an electrolyte with redox couple, and a counter electrode (cathode; CE), as shown in Figure 1b (generation III). The CE controls the entire DSSC function and regeneration reaction (Figure 2). Between the CE and the electrolyte interface, the critical regeneration reactions are illus-trated in Equations (1)–(5). The overall reactions of the electrochemical regeneration reactions are shown as Equation (1) and are divided into four steps. In the first step, decomposition (Equation (2)), the triiodide breaks into diiodine and iodide in the electrolyte. For iodide, it can diffuse to the photoanodic electrode for regenerating the oxidized dye. The diiodine formed in the second step, adsorption (Equation (3)), are separately adsorbed on the active sites of electrocatalyst. Subsequently, in the third step, the reduction reaction (Equation (4)), iodine is reduced to iodide on the active sites of electrocatalyst. In the final step, desorption, the reduced iodide is desorbed for the regenerating DSSC functions [12,13,14].

I_3_^−^ + 2e^−^ ↔ 3I^−^, (1)

I_3_^−^ ↔ I_2_ + I^−^, (2)

I_2_ + 2CE ↔ I (CE) + I (CE), (3)

I (CE) + e^−^↔I^−^ (CE), (4)

I^−^ (CE) ↔ I^−^ + CE, (5)

Platinum (Pt) is common, classic, and well-standard electro-catalyst for the CE of DSSCs with an I^−^/I_3_^−^—based electrolyte. As part of the development of DSSC, Pt could not satisfy the requirements for practical applications on an industrial scale because Pt is expensive and rare [15,16,17,18,19]. Furthermore, when DSSC is applied in other redox couples such as cobalt, copper, iron, pseudohalide and thiocyanate, Pt is not the best orthe most stable electro-catalyst. Therefore, it is important to find electro-catalysts to substitute in place of Pt. The ideal electro-catalyst offers advantages such as outstanding electro-catalytic ability, high redox couple stability, high electron conductive, natural abundance, low cost, and easy fabrication. According to suggestions, Pt-free electro-catalyst materials are targeted to carbonaceous materials, conducting polymers, transition metallic compounds, and their hybrid composites [19,20,21,22,23,24,25,26,27,28,29,30].

In this review, Pt-free electro-catalyst materials in recent DSSC applications are summarized to provide a better understanding and strategies to researchers. The extended Pt-free electro-catalyst materials usually have two aspects: one is the application of various materials, and the other is the nanostructure design. Therefore, their effects will be discussed individually with electrochemistry and physical and conversion efficiency.

## 2. Pt-Free Electro-Catalyst Materials

Pt-free electro-catalyst materials have two factors for increasing the performance of DSSCs, i.e., intrinsic electro-catalytic ability and nanostructure. The performance of electro-catalyst materials is judged by electrochemistry and conversion efficiency. Therefore, it is important to recognize the meaning of different parameters in electrochemistry and photoelectric conversion [12,13,17,31,32].

In electrochemistry, cyclic voltammetry (CV), Tafel polarization, and electrochemical impedance spectra (EIS) measurements are usually used to determine electro-catalytic performance. CV precisely quantifies the overall electro-catalytic ability and kinetic reduction capability of an electro-catalyst by two parameters: (1) the cathodic peak current density (*J_pc_*) and (2) the peak potential separation (Δ*E_p_*). The *J_pc_* is defined as the net peak current density from the cathodic current peak to the background curve. The Δ*E_p_* is defined as the potential difference between the anodic and cathodic current peaks. Generally, a larger *J_pc_* presents a better overall electrocatalytic ability. On the other hand, a lower Δ*E_p_* indicates a lower overpotential to trigger redox reaction [29,33].

EIS is used to acquire ohmic series resistance (*R_S_*) and the charge-transfer resistance (*R_ct-EIS_*) of electro-catalytic film in the Nyquist plot. Usually, EIS measurement is applied to the dummy cell system, which is a two-electrode system. It is also used for the study of electro-catalyst materials and their Nyquist plot, which shows two semicircles. In the high-frequency zone, the onset point of the first semicircle is ohmic series resistance (*R_S_*), which relates to the resistance between the substrate and electro-catalytic material. In the middle-frequency zone, the radius of the first semicircle is the charge transfer resistance (*R_ct-EIS_*), which refers to the charge transfer resistance between the electro-catalytic film and the electrolyte [5,34].

In photoelectric conversion, there are the overall solar-to-electrical energy conversion efficiency (*η*), the short-circuit current (*J_SC_*), open-circuit photovoltage (*V_OC_*), and the fill factor of the cell (*FF*). Commonly, electrocatalytic ability positively relates to the *J_SC_*, i.e., the better electrocatalytic ability shows larger *J_SC_*. The film formation and properties of electro-catalytic materials also obviously influence the values of *η*, *J_SC_*, *V_OC_*, and *FF* [3,35].

Next, we will discuss the various strategies and characteristics of carbon materials, conductive polymer materials, metal compound materials, and hybrids compound materials step-by-step.

### 2.1. Carbon Electro-Catalysts

Carbon materials have high electrical conductivity, good corrosion resistance, strong thermal stability, and adjustable energy levels. They are broadly classified into the following two types: (1) sp^3^-hybridization (e.g., amorphous porous carbon, carbon nanotube, carbon black, and activated carbon) and (2) sp^2^-hybridization (e.g., graphite, carbon nanotube, graphene, and fullerenes) [21,25,36,37,38,39]. However, carbon materials possess a smaller number of defective sites. To overcome this issue, the following three strategies are usually employed: (1) increasing the reaction surface area of carbon materials; (2) embedding the heteroatoms into the basal layers; (3) designing the specific electron pathway of electro-catalysts. Here, we list several pieces of literature with strategies for promoting the efficiency of DSSCs [2,21,23,37,40,41,42,43,44,45,46,47].

**Figure 3:**
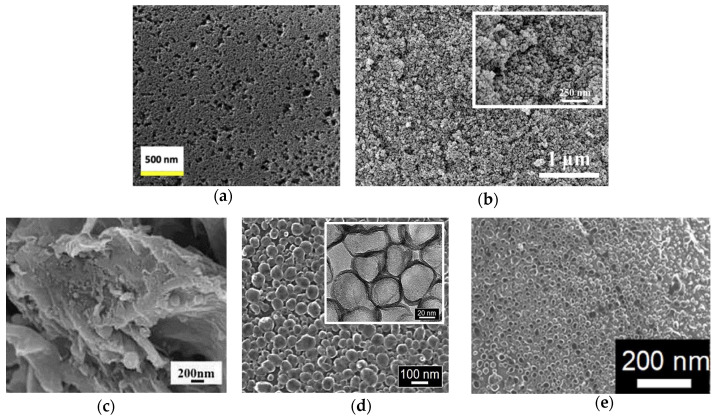
The SEM images of (**a**) HMC2021 [48], (**b**) CA-C [49], (**c**) N-CNOs/mGr [50], (**d**) N-GHBs (inset shows their TEM images) [52], and (**e**) N,S-GHBs [53].

Younas et al. obtained highly mesoporous carbon (HMC) through the adoption of the template method [48]. HMC2021 was grown with a 2:1 ratio of carbon precursor, and 20 nm colloidal silica shows mesoporosity and the greatest BET (Brunauer–Emmett–Teller) surface area among HMC412 (747 m^2^ g^−1^), HMC411 (947 m^2^ g^−1^), HMC1211 (628 m^2^ g^−1^), and HMC2021 (1037 m^2^ g^−1^). As shown in Figure 3a, the pore size and the diameter of the HMC2021 nanoparticles were 30–60 nm and ~20 nm, respectively. HMC2021 displayed the best *η* of 8.77% and the lowest *R_ct-EIS_* of 9 Ω cm^2^, which can be attributed to the large enhancement of electrocatalytic activity, as shown in Table 1 and Table 2. According to the results, the mesopore size and the surface area had a great correlation with electrocatalytic activity.

**Table 1:** The photovoltaic parameters of various carbon electrocatalysts in the DSSCs with an I^−^/I_3_^−^ redox couple and N719 dye are compared under 1.0 sun (AM 1.5G, 100 mW cm^−2^).

Sample	*η* (*%*)	*η* of *Pt* (*%*)	*J_SC_* (mA cm^−2^)	*V_OC_* (V)	*FF*	Surface Area *(*m^2^ g^−1^*)*	Ref.
HMC2021	8.77	7.57	16.10	0.81	0.68	1037	[48]
CA-C	9.08	7.92	16.59	0.77	0.71	724	[49]
N-CNOs/mGr	10.28	6.54	23.19	0.76	0.58	N/A	[50]
GD-1@hGO	9.10	8.80	16.00	0.74	0.78	353	[51]
N-GHBs/CC	7.53	7.70	16.09	0.70	0.67	N/A	[52]
N,S-GHBs/CC	9.02	8.90	15.71	0.80	0.72	N/A	[53]

**Table 2:** The electrochemical parameters of various carbon materials CEs under I^−^/I_3_^−^ redox couple.

Sample	*J_pc_* (mA cm^−2^)	Δ*E_p_* (V)	*R_ct-EIS_* (Ω cm^2^)	Ref.
HMC2021	N/A	N/A	9.00	[48]
CA-C	1.40	N/A	3.96	[49]
N-CNOs/mGr	1.61	0.56	2.30	[50]
GD-1@hGO	3.00	0.34	1.30	[51]
N-GHBs/CC	1.68	0.55	10.73	[52]
N,S-GHBs/CC	2.22	0.46	0.15	[53]

**Figure 4:**
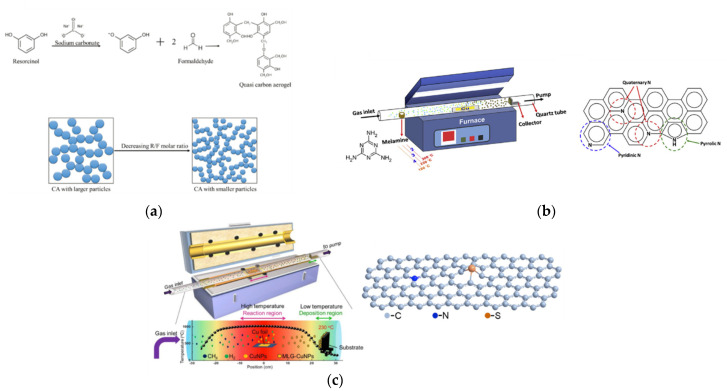
(**a**) Molecular equation of the additional reaction and the effect of the R/F molar ratios on the CA particles [49]. (**b**) Scheme of the CVD design for the growth of N-GHBs; three types of the N-doped states in N-GHBs include pyridinic N, pyrrolic N, and quaternary N [52]. (**c**) Scheme shows the growth of GHBs on CC in a CVD reactor at 1090 °C and the temperature profile inside the quartz tube of the CVD system [53].

Huang et al. synthesized pristine mesoporous carbon aerogels (CA) by means of various resorcinol (R)/formaldehyde (F) and resorcinol (R)/sodium carbonate (C) molar ratios, as shown in Figure 4a [49]. The R/F molar ratio and R/C ratio of CA-C was 377 and 0.76, respectively. Moreover, its specific surface area was up to 724 m^2^ g^−1^ and showed the smallest particle size of approximately 50 nm in diameter in the study (Figure 3b). The CA-C had the best *η* of 9.08%, the largest *J_pc_* of 1.40 mA cm^−2^, the lowest *R_ct-__EIS_* of 3.18 Ω cm^2^, and the smallest *R_ct-EIS_* of 3.96 Ω cm^2^ among the various CAs in the study, as shown in Table 1 and Table 2. Furthermore, the DSSC with the CA-C exhibitrf an impressive *η* of 20.1 ± 0.60% under a T5 lamp with 7000 lux (2.18 mW cm^−2^). Here, the specific surface areas could be increased by tuning the precursor ratio to enhance the electrocatalytic ability. Moreover, the DSSC with the CA-C demonstrates that the carbon electro-catalysts could be used in indoor solar cell applications.

Pang et al. synthesized N-doped carbon nano-onion (N-CNO) with modified graphene (mGr). The diameter of N-CNO and the thickness of mGr were 40–60 nm and 10–30 nm, respectively, as shown in Figure 3c [50]. The N-CNOs/mGr showed the best *η* of the 10.28% among the mGr (5.11%), and the Pt (6.54%), as shown in Table 1. In Table 2, N-CNOs/mGr shows an *J_pc_* of 1.61 mA cm^−2^ and a *R_ct-EIS_* of 2.30 Ω cm^2^, which are of better electrocatalytic ability than mGr (*J_pc_* of 1.61 mA cm^−2^ and *R_ct-EIS_* of 8.96 Ω cm^2^) and the Pt (*J_pc_* of 1.61 mA cm^−2^ and *R_ct-EIS_* of 0.15 Ω cm^2^). In the N-CNOs/mGr CEs, the N-CNOs could offer extra active sits for the reduction, and the mGr network benefitted intrinsic charge transfer.

Ali et al. acquired nitrogen-doped graphene quantum dots (NGQDs) from the hydrothermal cutting method with DMA adjustment (antisolvent) [51]. The GD-1@hGO morphology consisted of GD-1 (nanorod with 1 μm length and 3 μm width) and holey graphene oxide (hGO, nanosheet with irregular porous). In BET measurement, the GD-1@hGO, the hGO, and the rGO exhibited BET areas of 353, 135, and 60 m^2^ g^−1^, respectively. The GD-1@hGO presents the best *η* of 9.10% compared to the hGO (6.70%), the rGO (5.10%), and Pt (8.80%), as shown in Table 1. In electrochemical performance, the GD-1@hGO exhibited the largest *J_pc_* of 3.00 mA cm^−2^ and the lowest *R_ct-EIS_* of 1.30 Ω cm^2^ compared to the hGO (*J_pc_* of 1.61 mA cm^−2^ and *R_ct-EIS_* of 8.96 Ω cm^2^), the rGO (*J_pc_* of 1.61 mA cm^−2^ and *R_ct-EIS_* of 8.96 Ω cm^2^), and the Pt (*J_pc_* of 1.61 mA cm^−2^ and *R_ct-EIS_* of 8.96 Ω cm^2^), as shown in Table 2. The GD-1@hGO demonstrated more electrocatalytic activity, good electrolyte diffusion, and multidimensional charge transport channels.

Tseng et al. had grown nitrogen-doped graphene hollow nanoballs on carbon cloth (CC) (named N-GHBs) via the chemical vapor deposition (CVD) method, as shown in Figure 4b [52]. From SEM (Figure 3d) and TEM (inset of Figure 3d) images, the nanoball diameter of the N-GHBs was shown to be around 50–100 nm, and the thickness of the nanoball shell was about 3.5 nm, corresponding to approximately 10 layers of graphene. The catalytic activity of CC, GHBs, N-GHBs, and Pt were investigated along with photovoltaic parameters and IV curves in DSSCs; they are shown in Table 1. The N-GHBs reveals the best *η* among the CC (0.48%), the GHBs (6.20%), the N-GHBs (7.53%), and the Pt (7.70%). Moreover, the N-GHBs exhibited close electrocatalytic performance to Pt based on CV and EIS measurements, as shown in Table 2. These good results are contributed to by GHBs, which offer an increase of the specific surface area and the N-doped states in graphene to significantly enhance electrocatalytic activity.

Chang et al. grew heteroatoms-doped graphene hollow nanoballs (GHBs) on flexible carbon cloth (CC) by means of a CVD reaction with nitrogen (N) and sulfur (S) atoms, as shown in Figure 4c [53]. As seen in the SEM images (Figure 3e), the thickness and the diameter of the hollow nanoballs of N,S-GHBs were about 3.5 nm and 20–50 nm, respectively. Compared to various CEs in a DSSC, the N,S-GHBs showed the best *η* of 9.02% among the GHBs (6.47%), the N-GHBs (7.48%), the S-GHBs (8.15%), N,S-GHBs, and the Pt (8.90%), as shown in Table 1. In electrochemical performance, the N,S-GHBs still revealed the best electrocatalytic ability (*J_pc_* of 2.22 mA cm^−2^ and *R_ct-EIS_* of 0.15 Ω cm^2^), as shown in Table 2. In this study, the electrocatalytic ability of carbon electro-catalysts was the N,S-GHBs > the S-GHBs > the N-GHBs > the GHBs. These results indicate that the heteroatoms-doped strategy could be used to obviously boost the electrocatalytic ability.

In this carbon electro-catalyst section, there are three major strategies to enhance the DSSC performance: (1) creating active sites (e.g., N-doped carbon, S-doped carbon, etc.), (2) increasing specific surface areas via controlling precursors and template methods, and (3) constructing specific electron pathways using the specific structures and multidimensional structures. Accordingly, the disadvantages of the carbon electro-catalysts have been sufficiently overcome for holding a promising means to replace Pt in the future.

A brief summary of this section follows: Younas et al. developed a high-performance CE material (i.e., highly mesoporous carbon) by greatly enhancing the specific surface area for the electro-catalytic reaction. Additionally, Huang et al. utilized the same concept to boost the electro-catalytic ability of their Pt-free CE using mesoporous carbon aerogels. On the other hand, Pang et al. introduced the heteroatom-doped technology to increase the active sites of their carbon catalyst (i.e., N-doped carbon nano-onion) in conjunction with 2D-layered graphene (i.e., modified graphene) to facilitate the intrinsic charge transfer property. Further, Ali et al. used the same approach of heteroatom-doped technology to prepare nitrogen-doped graphene quantum dots composited with holey graphene oxide sheets for the use of Pt-free CE in DSSCs; where, the edge-site rich graphene quantum dots could provide more active site area for the electro-catalytic reaction compared to pristine 2D graphene materials (note: the basal plane of pristine graphene usually has poor activity for a electro-catalytic reaction). To address the issue of poor activity on the basal plane of pristine graphene, both the insertion of heteroatom into the basal plane of graphene and the structural engineering of 2D graphene to 3D shape/morphology approaches were adopted by Tseng et al. to effectively enhance the electro-catalytic ability of their graphene-based CE (i.e., N-doped graphene hollow nanoballs). Meanwhile, Chang et al. further improved the performance of the graphene-based CE based on the report of Tseng et al. via diatomic doping (N,S-doped graphene hollow nanoballs). Finally, the DSSCs with above carbon-based CEs almost exhibited superior cell performance than those of the cells with a Pt electrode.

### 2.2. Conductive Polymer Electro-Catalysts

**Figure 5:**
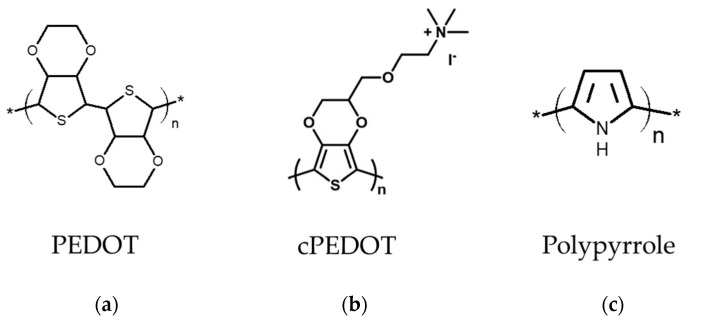
The molecular structures of the conductive polymer of (**a**) PEDOT [35], (**b**) cPEDOT [66], and (**c**) Polypyrrole [67].

Due to the flexible properties, good electrical conductivity, good adhesion to the substrates, and easy integration with roll-to-roll processes [31,54,55,56], several conductive polymer electro-catalysts such as PEDOT:PSS, PEDOT-MeOH, cPEDOT, PEDOT, PProDOT, PANI, and PPy are used in DSSCs. In this section, the PEDOT, cPEDOT, and PPPy (Figure 5) are chosen for further discussion because they have been demonstrated to perform well in various redox couples (Cu^2+^/Cu^+^ and I^−^/I_3_^−^), dyes (Y123, D35/XY1, XY1b/Y123, D149, and N719), light sources, and irradiation powers (10, 12, 50, 100 mW cm^−2^) [28,35,54,57,58,59,60,61,62,63,64,65,66,67]. Here, three strategies are listed to improve the performance of DSSCs with conductive polymer electro-catalysts: (1) the different dyes or electrolytes are allocated the DSSC with the conductive polymers; (2) the conductive polymers are linked to the specific functional group; (3) the reaction surface areas are increased in the conductive polymers.

**Figure 6:**
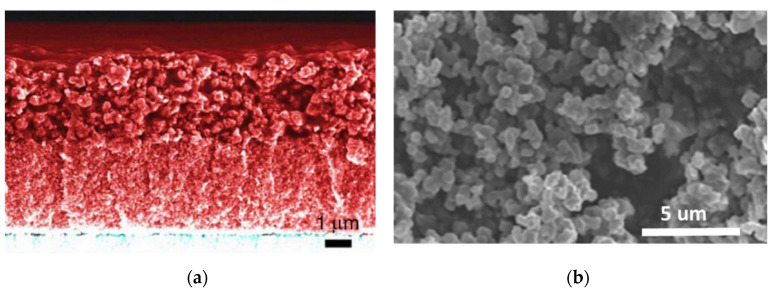
(**a**) The cross section SEM image of (**a**) a solid-state DSSC with PEDOT [35]. (**b**) The SEM image of porous polypyrrole [67].

Cao et al. synthesized the PEDOT on FTO glass, incorporating it with Y123 dye and solid-state Cu^+^/Cu^2+^ electrolytes for solid-state DSSC [35]. The PEDOT molecular structure and the solid-state DSSC cross section are shown in Figure 5a and Figure 6a, respectively. The solid-state DSSC shows the *η* of 11.00%, 11.30%, and 10.05% at the irradiation powers of 100, 50, and 10 mW cm^−2^, respectively, as shown in Table 3. For long-term stability testing, the *η* of solid-state DSSCs with the PEDOT is still up to 9.50%, even after a 35-day testing period. Due to the incorporation of Cu^+^/Cu^2+^ electrolytes and Y123 dye, the disadvantages of PEDOT CEs, such as instability and low electrocatalytic ability, could be eliminated, promising future for DSSC.

Freitag et al. obtained PEDOT films via the electrochemically deposited method, incorporating with D35/XY1 co-sensitizing dye and copper complex Cu(II/I)(tmby) electrolyte for fabricating DSSCs [63]. The DSSC reveals the *η* of 11.40% and 13.20% at an irradiation power of 100 and 12 mW cm^−2^, respectively, as shown in Table 3. The DSSC performance is increased with decreasing irradiation power, indicating that DSSC can be well used in various weather conditions such as on sunny, cloudy, and rainy days. Furthermore, the *η* of the DSSCs are 28.90% and 25.50% under indoor-light illumination at a power of 0.306 and 0.061 mW cm^−2^. These results support the DSSC for use in various irradiation conditions with different light sources.

**Figure 7:**
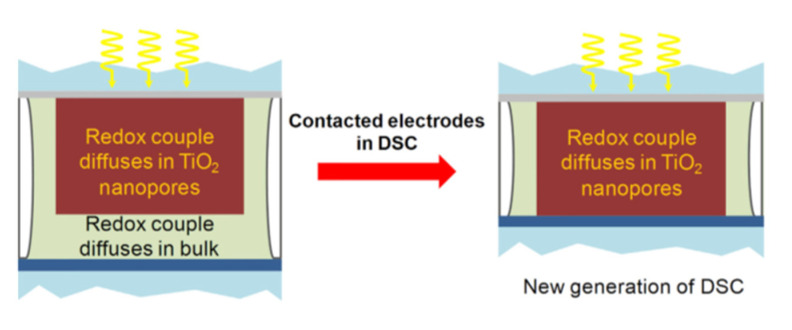
Schematic of the new DSSC architecture employing electron-blocking p-type hole-specific charge collectors in direct contact with the mesoporous TiO_2_ scaffold [65].

Cao et al. deposited PEDOT on FTO glass through the electrochemical deposition method to prepare a solid-state DSSC [65]. The solid-state DSSC consisted of working electrodes with XY1b and Y123 dyes, solid-state Cu^+^/Cu^2+^ electrolytes, and PEDOT CE, as shown in Figure 7. The DSSC shows the *η* of 13.10% at solar simulation power of 100 mW cm^−2^ in Table 3. Moreover, the *η* of the DSSC are 31.80%, 30.80%, and 27.50% under indoor-light illumination with irradiation powers of 0.318, 0.159, and 0.063 mW cm^−2^.

Bella et al. synthesized the poly(3,4-ethylenedioxythiophene) derivative bearing a cationic ammonium moiety with an iodide counter-anion (cPEDOT) through a wet chemical method; the cPEDOT CE was fabricated via spin coating on the FTO substrate [66]. The DSSC using cPEDOT CE and a 100% aqueous electrolyte based on the I^−^/I_3_^−^ redox couple presents the *η* of 7.02%, which is better than the *η* of *Pt* (5.38%) and PEDOT:PSS (3.91%), as shown in Table 3. In terms of electrochemical performance, cPEDOT shows a larger *J_pc_* of 0.76 mA cm^−2^ and a lower *R_ct-EIS_* of 3.29 Ω cm^2^, which is lower than that of Pt (*J_pc_* of 0.61 mA cm^−2^ and lower *R_ct-EIS_* of 5.33 Ω cm^2^), as shown in Table 4. In addition, the cPEDOT and Pt could still maintain 96% and 94% of their initial efficiency after 1200 h under simulated sunlight, indicating the long-term stability. The outstanding stability and performance of DSSC are contributed by the conductive polymers electro-catalyst bonded to specific functional groups, e.g.*,* the ammonium group, hydroxyl group, poly(styrene sulfonate), etc.

Khan et al. acquired porous polypyrrole (PPPy) by using the hydrothermal method with ZIF-8 at different temperatures (60, 80, 100, and 120 °C), and then the ZIF8 could be removed using a HCl solution (pH = 4) [67]. The nanoparticle diameter of porous polypyrrole @100 °C was around 50–70 nm, as shown in Figure 6b. Moreover, the specific surface area of the porous polypyrrole @60 °C, @80 °C, @100 °C, and @120 °C are 125.04, 268.64, 323.12, and 30.64 m^2^ g^−1^, respectively. The porous polypyrrole @ 100 °C shows the best *η* of 8.63% compared to Pt (9.05%), the CPPy (5.20%), the porous polypyrrole @60 °C (7.18%), the porous polypyrrole @80 °C (8.13%), and the porous polypyrrole @120 °C (6.72%), as shown in Table 3. Furthermore, the porous polypyrrole @100 °C exhibits the larger *J_pc_* (0.76 mA cm^−2^) and lower *R_ct-EIS_* (3.29 Ω cm^2^) than Pt (*J_pc_* of 0.76 mA cm^−2^ and *R_ct-EIS_* of 3.29 Ω cm^2^), the CPPy (*J_pc_* of 0.76 mA cm^−2^ and *R_ct-EIS_* of 3.29 Ω cm^2^), the porous polypyrrole @60 °C (*J_pc_* of 0.76 mA cm^−2^ and *R_ct-EIS_* of 3.29 Ω cm^2^), the porous polypyrrole @80 °C (*J_pc_* of 0.76 mA cm^−2^ and *R_ct-EIS_* of 3.29 Ω cm^2^), and the porous polypyrrole 120 °C (*J_pc_* of 0.76 mA cm^−2^ and *R_ct-EIS_* of 3.29 Ω cm^2^), as shown in Table 4. According to these results, DSSC performance could be enhanced effectively by increasing the specific surface area.

Accordingly, DSSC performance could be improved by the following strategies: (1) collocating the various dye and redox couples, (2) the polymers electro-catalysts bonding functional groups, and (3) increasing the specific surface area, leading to the applications of DSSCs under various light sources and light intensities.

A brief summary of this section follows: Freitag et al. used an electro-deposited PEDOT CE in conjunction with home-made sensitizers and copper complex Cu(II/I)(tmby) electrolytes for the fabrication of DSSCs, which reached an efficiency record of 11.40% for the Pt-free DSSCs without using the traditional I^−^/I_3_^−^—based electrolytes. In the same group, Cao et al. developed a highly efficient solid-state DSSC exhibiting an impressive efficiency record of 13.10% by using an electro-deposited PEDOT CE in conjunction with home-made sensitizers and solid-state Cu^+^/Cu^2+^ electrolyte. Their solid-state DSSC even showed an amazing efficiency of 31.80% under indoor-light illumination (0.318 mW cm^−2^) and a unfailing long-term stability. Bella et al. developed a Pt-free DSSC using a cPEDOT (i.e., poly(3,4-ethylenedioxythiophene) derivative bearing a cationic ammonium moiety with an iodide counter-anion) CE and a 100% aqueous electrolyte based on the I^−^/I_3_^−^ redox couple, which presented a cell efficiency higher than that of a cell with Pt CE. Most importantly, this report is the first example of a DSSC device able to avoid the use of organic solvent-based electrolytes, platinum, cobalt, and ruthenium while outperforming the devices assembled with one or more of these heavy/rare metals. Another type of conductive polymer, i e., polypyrrole (PPy), was also employed as an electro-catalyst in the CE of the DSSCs; the PPy CE possesses a porous structure, which was constructed using zeolitic imidazolate framework-8 (ZIF-8) as template via the simple hydrothermal method.

### 2.3. Transition Metallic Compound Electro-Catalysts

Metal compound electro-catalysts fascinate scientists because their electron orbitals are similar to Pt [16,68,69]. In other words, metal compound electro-catalysts show a lot of potential for replacing Pt as the electro-catalysts. The metal compound electro-catalysts include carbides, nitrides, chalcogenides, oxides, phosphides, and so on [69,70,71]. In this section, the partial reports also studied the performance of DSSCs using metal compound electro-catalysts under lower solar irradiation power and indoor-light sources. The metal compound electro-catalysts could improve the performance of the DSSCs by (1) using the various compounds, (2) incorporating with different redox couples, (3) increasing the reaction surface areas, and (4) creating specific electron pathways [16,20,22,24,72,73,74,75,76,77]. Here, several pieces of literature about the efficiency improvement of DSSC by metal compound electro-catalysts will be briefly introduced.

**Figure 8:**
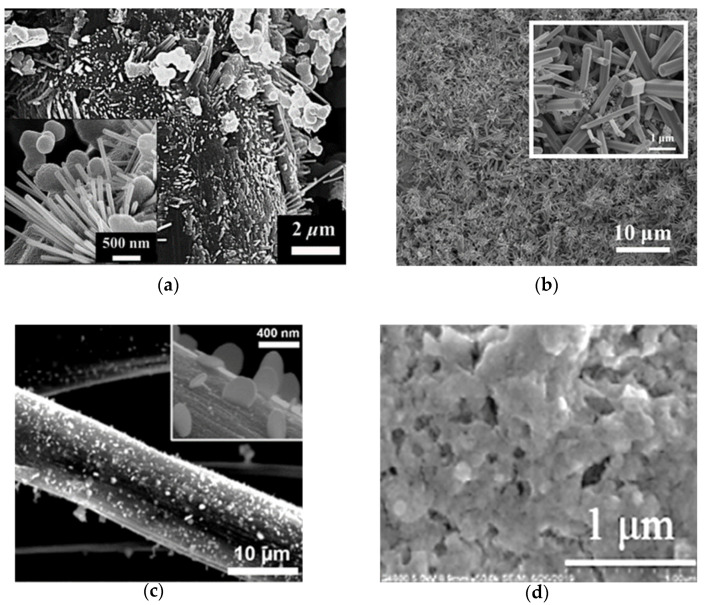
The SEM images of (**a**) TiO_1.1_Se_0.9_/CC [78], (**b**) CoSe_2_/CoSeO_3_-UL [79], (**c**) T_d_-Mo_0.29_W_0.72_Te_1.99_/CC [80], and (**d**) CIS-10 [81].

**Figure 9:**
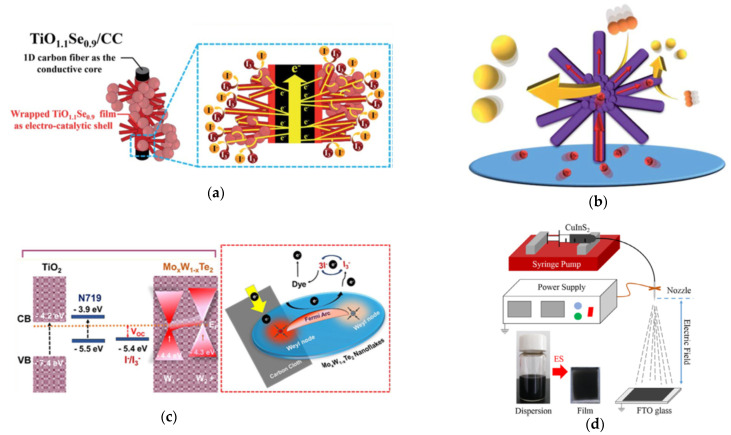
(**a**) The core–shell sketch of the electrodes of TiO_1.1_Se_0.9_/CC and its hierarchical electron transfer route for executing the reduction of I_3_^−^ ions [78]. (**b**) The sketch of the electron transport route of CoSe_2_/CoSeO_3_-UL [79]. (**c**) A schematic representation of the topological Weyl semi-metal (TWS) surface of a T_d_-Mo_0.29_W_0.72_Te_1.99_/CC [80]. (**d**) Schematic diagram of the electrospray process and the photographs of the CIS dispersion solution and CIS films [81].

Li et al. synthesized the TiO_1.1_Se_0.9_/CC by means of the wet chemical method [78]. The TiO_1.1_Se_0.9_/CC composites of nanospheres (500 nm diameter) and nanorod (2 μm length and 50 nm diameter) are as shown in Figure 8a. The DSSC with the TiO_1.1_Se_0.9_/CC presented the *η* of 9.47%, which is better than the Pt/CC (7.75%) and the TiO_2_/CC (4.90%), as shown in Table 5. Furthermore, the *η* of TiO_1.1_Se_0.9_/CC using the I^−^/I_3_^−^ and Cu^+^/Cu^2+^ electrolytes are measured at 1.0 sun (100 mW cm^−2^), 0.5 sun (50 mW cm^−2^), and 0.1 sun (10 mW cm^−2^). Using the iodide-based electrolyte, the *η* of TiO_1.1_Se_0.9_/CC are 9.47% at full sunlight (1.0 sun), 10.00% at medium light (0.5 sun), and 10.39% (the best one) at dim light (0.1 sun). Using the Co^+^/Co^2+^ electrolyte, the *η* of TiO_1.1_Se_0.9_/CC are 10.32% at 1.0 sun, 10.47% (the best one) at 0.5 sun, and 10.20% at 0.1 sun. In Table 6, the TiO_1.1_Se_0.9_/CC exhibits the larger *J_pc_* (8.16 mA cm^−2^) and lower *R_ct-EIS_* (1.21 Ω cm^2^) than the Pt/CC (*J_pc_* of 5.67 mA cm^−2^ and *R_ct-EIS_* of 3.28 Ω cm^2^) and the TiO_2_/CC (*J_pc_* of 1.25 mA cm^−2^ and *R_ct-EIS_* of 9.38 Ω cm^2^). The DSSC performance is enhanced by hierarchical structure (offering a hierarchical electron transfer route) and using the cobalt redox couples, as shown in Figure 9a, leading to the high potential of TiO_1.1_Se_0.9_/CC in the dim light applications.

Huang et al. obtained the hierarchical urchin-like CoSe_2_/CoSeO_3_-UL (CoSe_2_/CoSeO_3_-UL) via the hydrothermal method [79]. Figure 8b reveals the assemble nanoparticles (50 nm diameter) and extended nanorods (1–3 mm length and about 100–500 nm diameter) of the CoSe_2_/CoSeO_3_-UL. The CoSe_2_/CoSeO_3_-UL exhibits a *η* of 9.29%, which is better than the Pt (8.33%) and the CoSe_2_/CoSeO_3_-NP (8.81%), as shown in Table 5. In the dim light environment, the CoSe_2_/CoSeO_3_-UL CE shows impressive *η* of 19.88%, 18.24%, and 16.00% at 7000 lux (2.21 mW cm^−2^), 6000 lux (1.89 mW cm^−2^), and 4800 lux (1.55 mW cm^−2^), respectively. For electrochemical performance, the CoSe_2_/CoSeO_3_-UL expresses the largest *J_pc_* of 1.90 mA cm^−2^ and the lowest *R_ct-EIS_* of 0.99 Ω cm^2^ compared to Pt (*J_pc_* of 1.10 mA cm^−2^ and *R_ct-EIS_* of 2.02 Ω cm^2^), CoSe_2_/CoSeO_3_-UL, and CoSe_2_/CoSeO_3_-NP (*J_pc_* of 1.51 mA cm^−2^ and *R_ct-EIS_* of 1.88 Ω cm^2^), as shown in Table 6. In this case, the hierarchical UL structure provides a high surface area for catalytic reactions and a one-dimensional (1D) charge transport route, as shown in Figure 9b.

Hudie et al. acquired a semi-metallic Mo_x_W_1−x_Te_2_ nanosheet on CC (T_d_-Mo_0.29_W_0.72_Te_1.99_/CC) by means of the CVD method [80]. Figure 8c shows the vertical nanosheet (300–400 nm) structures of the T_d_-Mo_0.29_W_0.72_Te_1.99_/CC. The *η* (8.85%) of T_d_-Mo_0.29_W_0.72_Te_1.99_/CC is better than the Pt/CC (*η* of 8.01%), as shown in Table 5. Furthermore, the T_d_-Mo_0.29_W_0.72_Te_1.99_/CC shows larger *J_pc_* (2.72 mA cm^−2^) and lower *R_ct-EIS_* (0.21 Ω cm^2^) than the Pt/CC (*J_pc_* of 2.03 mA cm^−2^ and *R_ct-EIS_* of 0.49 Ω cm^2^), as shown in Table 6. The emerging Weyl semi-metals with robust topological surface states and vertical nanosheet structures could offer the low charge-transfer resistance (Figure 9c) and vertical electron pathways, leading to the higher electrocatalytic ability of the T_d_-Mo_0.29_W_0.72_Te_1.99_/CC.

Xu et al. synthesized CuInS_2_ (CIS) films by means of the electrospray method with different electrospray times (5, 10, and 15 min) and heat treatment, as shown in Figure 9d [81]. The SEM images reveal the porous network structures of CuInS_2_ with 10 min electrospray (CIS-10) (Figure 8d), which is the continuity of nanoparticles and the interconnectivity of the pore channels. DSSC with CIS-10 exhibits a *η* of 8.81%, which is better than CIS-5 (*η* of 7.72%), CIS-15 (*η* of 6.02%), and Pt (*η* of 7.77%), as shown in Table 5. For the electrochemical measurements, CIS-10 presents the highest *J_pc_* and the smallest *R_ct-EIS_* compared to CIS-5 (*J_pc_* of 1.98 mA cm^−2^ and *R_ct-EIS_* of 9.16 Ω cm^2^), CIS-10 (*J_pc_* of 2.33 mA cm^−2^ and *R_ct-EIS_* of 5.18 Ω cm^2^), CIS-15 (*J_pc_* of 1.61 mA cm^−2^ and *R_ct-EIS_* of 8.81 Ω cm^2^), and Pt (*J_pc_* of 2.29 mA cm^−2^ and *R_ct-EIS_* of 6.22 Ω cm^2^), as shown in Table 6. In this case, the more active sites and diffusion channels are provided by the bimetal and the porous morphology, respectively.

Baptayev et al. synthesized CuCo_2_S_4_ nanoflowers via the solvothermal method at a low temperature [82]. The CuCo_2_S_4_ nanoflowers show a diameter of 8–9 μm and a height of around 5 μm. Furthermore, the BET surface area of CuCo_2_S_4_ nanoflowers is about 36.99 m^2^ g^−1^ with dominating pore sizes of 3.72 nm, indicating the mesoporous nature of CuCo_2_S_4_ nanostructures. The CuCo_2_S_4_ reveals the *η* (7.56%), which is comparable to Pt (7.42%), as shown in Table 5. The CuCo_2_S_4_ also exhibits *R_ct-EIS_* (24.2 Ω cm^2^) that is comparable to Pt (32.1 Ω cm^2^), as shown in Table 6. Here, the multiple transition metal compounds and the remarkably high surface areas of the nanoflowers are employed to enhance the DSSC’s electrocatalytic ability and performance.

In this metal compound’s electro-catalyst section, the four strategies used to increase the performance of the DSSCs are briefly introduced: (1) using different redox couples, (2) combining multiple transition metal compounds, (3) increasing high surface areas, and (4) offering more sufficient electron pathways. The above results demonstrate that the metal compound electro-catalysts hold promise for next-generation solar cells for operation in low solar power and indoor dim light conditions.

A brief summary on this section follows: Both Li et al. and Huang et al. (the same group) developed transition metal oxide/chalcogenide composite electro-catalysts (i.e., TiO_1.1_Se_0.9_ and CoSe_2_/CoSeO_3_, respectively) with hierarchical nanostructures as highly efficient CEs for Pt-free DSSCs, where the hierarchical nanostructure consisted of nanoparticles and nanorods providing a high surface area and a 1D route for the electro-catalytic reaction and charge transport, respectively. Different to above two reports, Hudie et al., Xu et al., and Baptayev et al. synthesized bimetal chalcogenides as highly efficient electro-catalysts (i.e., T_d_-Mo_0.29_W_0.72_Te_1.99_ nanosheet, CuInS_2_ (CIS) film, and CuCo_2_S_4_ nanoflower, respectively) for Pt-free DSSCs. Notably, among them, the T_d_-Mo_0.29_W_0.72_Te_1.99_ nanosheet possessed the characterization of a topological Weyl semi-metal, which would greatly promote the rapid charge transfers as well as the high electro-catalytic activity. Finally, the DSSCs with the above transition metallic compound-based CEs almost exhibited superior cell performance than that of the cells with a Pt electrode.

### 2.4. Hybrid Compounds Electro-Catalysts

**Figure 10:**
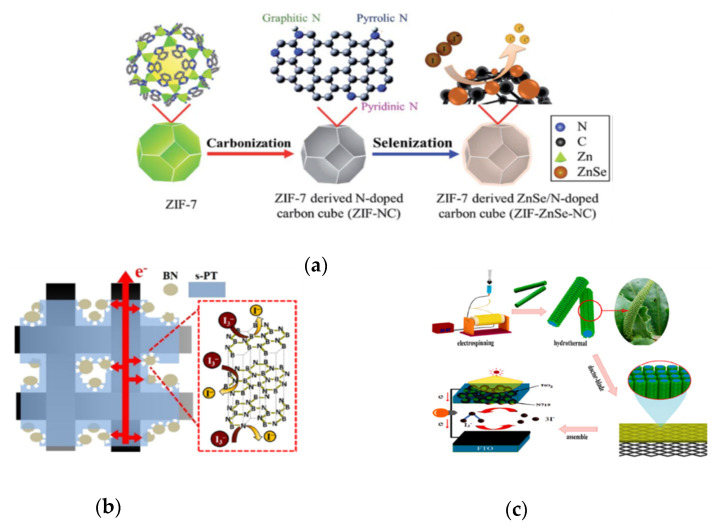
(**a**) The sketch of the process from ZIF-7 to ZIF-derived materials [86]. (**b**) Sketch of the electron transfer phenomenon in BN/s-PT-50 electrode [87]. (**c**) The fabrication process of a dye-sensitized solar cells with the Bi_2_MoO_6_/CNFs composite [89].

Hybrid compound electro-catalysts based on carbon, conductive polymer, and metal compound electro-catalysts could offer some advantages such as good electrocatalytic ability, good corrosion resistance, adjustable energy levels, good electrical conductivity, good adhesion to the substrate, and high possibility of roll-to-roll processing [3,13,17,18,19,23,83,84,85]. Here, some literature about the hybrid compounds in DSSC improvements based on the following four concepts are briefly introduced: (1) combining different material compounds, (2) increasing the reaction surface areas, (3) embedding heteroatoms into basal layers, and (4) increasing specific electron pathways (Figure 10).

**Figure 11:**
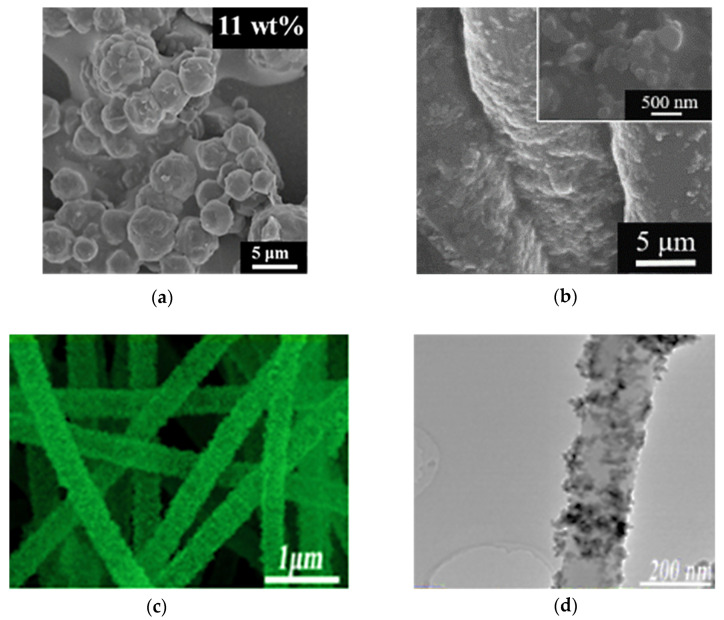
SEM images of (**a**) ZIF-ZnSe-NC-11 wt% [86], (**b**) BN/s-PT-50 [87], and (**c**) Bi_2_MoO6/CNFs [89]. (**d**) The TEM image of Bi_2_MoO_6_/CNFs [89].

Jian et al. produced the ZnSe/N doped carbon (ZIF-ZnSe-NC) cube hybrid electrocatalyst derived from zeolitic imidazolate framework by carbonization and selenization, as shown in Figure 10a [86]. The particle size of the ZIF-ZnSe-NC-11 wt% was ~3–6 μm, as shown in Figure 11a. ZIF-ZnSe-NC-11 wt% had the best performance compared to the ZIF-7 (5.27%), the ZIF-7-NC (6.02%), the ZIF-ZnSe-NC-11 wt% (8.69%), and the Pt (8.26%), as shown in Table 7. Furthermore, the ZIF-ZnSe-NC-11 wt% showed *η* of 7.99%, 8.02%, and 8.69% under 10, 50 and 100 mW cm^−2^ illumination, respectively, as shown in Table 7. For electrochemical properties (Table 8), the ZIF-ZnSe-NC-11 wt% could offer a *J_pc_* that was larger (0.91 mA cm^−2^) and a *R_ct-EIS_* that was lower (1.26 Ω cm^2^) than the Pt (*J_pc_* of 0.85 mA cm^−2^ and *R_ct-EIS_* of 1.71 Ω cm^2^). In this study, the good performance of the DSSCs is contributed to (1) the excellent electrocatalytic properties of ZnSe, (2) more active sites induced by N-doped carbons, and (3) the electrocatalytic ability enhanced by ZIF-7.

**Table 7:** The photovoltaic parameters of various conductive polymer material-based CEs in the DSSC with an I^−^/I_3_^−^ redox couple and N719 dye are compared under simulated solar light conditions.

Sample	*η* (*%*)	*η* of *Pt* (*%*)	*J_SC_* (mA cm^−2^)	*V_OC_* (V)	*FF*	P_in_ (mW cm^−2^)	Ref.
ZIF-ZnSe-NC-11 wt%	8.69	8.26	16.40	0.77	0.69	100	[86]
	8.02	7.87	7.60	0.73	0.72	50	
	7.99	7.41	1.63	0.68	0.72	10	
BN/s-PT-50	9.21	8.11	16.59	0.78	0.71	100	[87]
Fe_3_O_4_/Ni@N-RGO	8.96	7.87	16.50	0.78	0.70	100	[88]
Bi_2_MoO_6_/CNFs	9.02	7.47	14.78	0.84	0.73	100	[89]

**Table 8:** The electrochemical parameters of various hybrid compound electrocatalysts measured in the I^−^/I_3_^−^-based electrolyte.

Sample	*J_pc_* (mA cm^−2^)	Δ*E_p_* (V)	*R_ct-EIS_*(Ω cm^2^)	Ref.
MOF-525/s-PT-3	2.03	0.61	1.42	[29]
ZIF-ZnSe-NC-11 wt%	0.91	0.52	1.26	[86]
BN/s-PT-50	6.88	0.68	1.06	[87]
Fe_3_O_4_/Ni@N-RGO	4.44	0.19	0.16	[88]
Bi_2_MoO_6_/CNFs	3.03	0.44	0.61	[89]

Chen et al. obtained a boron nitride/sulfonated poly(thiophene-3-[2-(2-methoxyethoxy)ethoxy]-2,5-diyl) (denoted as BN/s-PT) composite electrocatalyst by means of physical mixing [87]. The BN/s-PT-30, BN/s-PT-40, BN/s-PT-50, BN/s-PT-60, and BN/s-PT-70 contained the BN/s-PT with a weight percentage of 30, 40, 50, 60, and 70, respectively. The SEM images (Figure 11b) of the BN/s-PT-50 show the BN nanoparticles (500 nm) covered by the s-PT polymer. BN/s-PT-50 shows the best *η* of 9.21% compared to the BN/s-PT-30 (7.46%), the BN/s-PT-40 (8.26%), the BN/s-PT-60 (8.22%), the BN/s-PT-70 (7.02%) and the Pt (8.11%), as shown in Table 7. Moreover, as the BN/s-PT-50 was operated in an indoor environment, the *η* was 21.02%, 19.52%, and 17.48% at 1.94 (6000), 0.98 (3000), and 0.33 mW cm^−2^ (1000 lux), respectively. As shown in Table 8, BN/s-PT-50 exhibited the best electrocatalytic properties (*J_pc_* of 6.88 mA cm^−2^ and *R_ct-EIS_* of 1.06 Ω cm^2^). In this study, BN offered more active sites for electrochemical reactions, and s-PT supported adhesion and conducting, as shown in Figure 10b.

Xu et al. acquired Fe_3_O_4_/Ni@N-RGO nanoflowers from the hydrothermal method [88]. The Fe_3_O_4_/Ni@N-RGO nanoflowers show an average size of about 2 μm and thin nanoflakes with numerous pores. The *η* (8.96%) of Fe_3_O_4_/Ni@N-RGO was better than Fe_3_O_4_ (7.92%), Fe_3_O_4_/Ni (8.25%), Fe_3_O_4_/Ni@RGO (8.53%), and Pt (7.87%), as shown in Table 7. For electrochemical properties, the *J_pc_* (4.44 mA cm^−2^ )and *R_ct-EIS_* (0.16 Ω cm^2^) of Fe_3_O_4_/Ni@N-RGO were also better than Fe_3_O_4_ (3.02 mA cm^−2^ and *R_ct-EIS_* of 0.49 Ω cm^2^), Fe_3_O_4_/Ni (3.23 mA cm^−2^ and *R_ct-EIS_* of 0.21 Ω cm^2^), Fe_3_O_4_/Ni@RGO (4.02 mA cm^−2^ and *R_ct-EIS_* of 0.17 Ω cm^2^), and Pt (2.81 mA cm^−2^ and *R_ct-EIS_* of 0.51 Ω cm^2^), as shown in Table 8, indicating the better electrocatalytic properties. The DSSC performance could be improved by (1) the more reaction areas and electron pathways offered by hierarchical porous Fe_3_O_4_ nanoflowers decorated with Ni nanoparticles and RGO nanosheets, (2) more active sites provided by heteroatoms embedded into graphene oxide, and (3) the excellent electrocatalytic properties of Fe_3_O_4_, Ni, and RGO.

Li et al. prepared the carbon nanofibers supported Bi_2_MoO_6_ nanosheets (Bi_2_MoO_6_/CNFs) by electrospinning and hydrothermal methods, as shown in Figure 10c [89]. The thickness of Bi_2_MoO_6_ nanosheets was 10–20 nm, and the diameter of the CNFs was 400–500 nm, as shown in Figure 11c,d. Moreover, the specific surface of Bi_2_MoO_6_/CNFs and the CNFs were 32.81 and 18.82 m^2^ g^−1^, respectively. The Bi_2_MoO_6_/CNFs exhibited the best performance compared to the Bi_2_MoO_6_/CNFs (9.02%), the CNFs (7.48%), and the Pt (7.487%), as shown in Table 7. According to Table 8, the *J_pc_* (3.03 mA cm^−2^) and *R_ct-EIS_* (0.61 Ω cm^2^) of Bi_2_MoO_6_/CNFs are better than the CNFs (2.76 mA cm^−2^ and *R_ct-EIS_* of 1.31 Ω cm^2^), and the Pt (2.00 mA cm^−2^ and *R_ct-EIS_* of 2.32 Ω cm^2^). These results reveal that Bi_2_MoO_6_ provides more active sites and that CNFs support better electrical conductivity for better DSSC performance.

In this hybrid compound electro-catalyst section, the strategies for improving DSSC performance can be summarized as follows: (1) selecting large surface area materials, (2) using a facilitating conducting material, (3) assembling composite materials, and (4) designing specific morphology. Accordingly, the as-fabricated DSSCs could hold promise for applications under dim light or various weather conditions as well as in indoor environments.

A brief summary of this section follows: Jian et al. proposed a new concept for preparing a ZnSe/N-doped carbon cube hybrid electrocatalyst via the carbonization and selenization of ZIF-7 (i.e., zeolitic imidazolate framework) for the first time, where the N-doped carbon cube is beneficial to the electrocatalytic performance and the electrical conductivity, and the embedded ZnSe in the carbon matrix serves as the additional active site for facilitating I_3_^−^ reduction. Chen et al. prepared the BN/s-PT composite electrocatalyst for a Pt-free DSSC, obtaining a significantly enhanced cell efficiency. This achievement is due to the synergetic effect of the BN nanoparticle and the s-PT binder; the former offered a large active surface area and a high intrinsic heterogeneous rate constant, and the latter formed fast electron transfer matrices. Xu et al. designed and synthesized a novel nanostructured composite electro-catalyst (i.e., three-component composite) that consisted of hierarchical porous Fe_3_O_4_ nanoflowers decorated with Ni nanoparticles and wrapped with N-doped reduced graphene oxide nanosheets (denoted Fe_3_O_4_/Ni@N-RGO), where the hierarchically nanostructure of flower-like Fe_3_O_4_ could offer a 3D porous scaffold with a large specific surface area for loading Ni nanoparticles and N-doped graphene nanosheets; thus, the as-obtained Fe_3_O_4_/Ni@N-RGO composite can afford high catalytic activity, excellent electrical conductivity, and abundant nanopores to interact with the I_3_^−^ ions. Li et al. successfully prepared Bi_2_MoO_6_/CNFs composites by electrospinning and hydrothermal methods, where the carbon material (i.e., CNFs) had good electrical conductivity and where the transition metal oxides (i.e., Bi_2_MoO_6_) could provide more active sites for better catalytic performance. Finally, the DSSCs with the above hybrid compound-based CEs exhibited superior cell performance than that of the cell with a Pt electrode.

## 3. Conclusions

So far, Pt-free electro-catalytic materials, e.g., carbonaceous materials, conducting polymers, transition metallic compounds, and their hybrid composites, have been successfully developed as the highly efficient counter electrodes for DSSCs, and most of them even show better electro-catalytic performance than Pt. The DSSCs with these Pt-free electro-catalytic electrodes thus exhibited superior cell performance than that of the cells with a Pt electrode. This achievement owes much to the structural (i.e., low-dimensional nanostructure design or atomic and molecule level design) and interface engineering (i.e., heteroatom-doped approach) conducted on these Pt-free electro-catalytic materials as well as the synergy effects that come from each material in their composites. At this stage, the concurrent advantage in high performance and low-cost materials for the DSSCs using these Pt-free counter electrodes can allow the promising future of DSSCs for mass production. The aforementioned advantages would also make the Pt-free electro-catalyst a promising electrode material for a wide variety of electrochemical applications, such as water electrolysis, supercapacitor, Li-battery, fuel cell, and sensing, etc. For the future development of Pt-free electro-catalytic electrodes, economical synthesis approaches toward both green and roll-to-roll processes could be the key issue that renders DSSCs being more competitive in the solar cell markets.

## Figures and Tables

**Table 3:** The photovoltaic parameters of various conductive polymer-based electrocatalysts in the DSSC are compared under simulated solar light conditions.

Sample	*η* (*%*)	*η* of *Pt* (*%*)	*J_SC_* (mA cm^−2^)	*V_OC_* (V)	*FF*	Redox Couple	Dye	P_in_ (mW cm^−2^)	Ref.
PEDOT	11.00	N/A	13.87	1.08	0.73	Cu^2+^/Cu^+^	Y123	100	[35]
	11.30		7.00	1.06	0.76			50	
	10.50		1.40	1.01	0.75			10	
PEDOT	11.30	N/A	16.19	1.03	0.68	Cu^2+^/Cu^+^	D35/XY1	100	[63]
	13.20		2.17	0.96	0.78			12	
PEDOT	13.10	N/A	15.74	1.05	0.79	Cu^2+^/Cu^+^	XY1b/Y123	100	[65]
cPEDOT	7.02	5.38	12.41	0.69	0.77	I^−^/I_3_^−^	D149	100	[66]
PPPy	8.63	9.05	17.03	0.71	0.71	I_4_^−^/I_3_^−^	N719	100	[67]

**Table 4:** The electrochemical parameters of various conductive polymer electrocatalysts under I^−^/I_3_^−^ redox couple.

Sample	*J_pc_* (mA cm^−2^)	Δ*E_p_* (V)	*R_ct-EIS_* (Ω cm^2^)	Ref.
cPEDOT	0.76	0.34	3.29	[66]
PPPy	0.62	0.68	2.60	[67]

**Table 5:** The photovoltaic parameters of various metal compound electrocatalysts in the DSSC with an I^−^/I_3_^−^ redox couple are compared under 1.0 sun (AM 1.5G, 100 mW cm^−2^).

Sample	*η* (*%*)	*η* of *Pt* (*%*)	*J_SC_* (mA cm^−2^)	*V_OC_* (V)	*FF*	Ref.
TiO_1.1_Se_0.9_/CC	9.47	7.75	17.22	0.79	0.70	[78]
CoSe_2_/CoSeO_3_-UL	9.29	8.33	16.09	0.82	0.70	[79]
T_d_-Mo_0.29_W_0.72_Te_1.99_/CC	8.85	8.01	15.44	0.78	0.73	[80]
CIS-10	8.81	7.77	22.90	0.79	0.49	[81]
CuCo_2_S_4_	7.56	7.42	15.99	0.74	0.64	[82]

**Table 6:** The electrochemical parameters of various metal compound electrocatalysts measured in the I^−^/I_3_^−^-based electrolyte.

Sample	*J_pc_* (mA cm^−2^)	Δ*E_p_* (V)	*R_ct-EIS_*(Ω cm^2^)	Ref.
TiO_1.1_Se_0.9_/CC	8.16	0.61	1.21	[78]
CoSe_2_/CoSeO_3_-UL	1.90	0.46	0.99	[79]
T_d_-Mo_0.29_W_0.72_Te_1.99_/CC	2.72	0.43	0.21	[80]
CIS-10	2.33	0.48	5.18	[81]
CuCo_2_S_4_	N/A	N/A	24.2	[82]
